# How Likely Do Patients After Total Knee Arthroplasty with a Posterior-Stabilized Knee System Meet Their Desired Sport Activity Level?

**DOI:** 10.3390/jcm15031255

**Published:** 2026-02-04

**Authors:** Tobias Scheidl, Oliver Haider, Martin Faschingbauer, Christian Manuel Sterneder, Friedrich Boettner, Maximilian F. Kasparek

**Affiliations:** 1 Vienna General Hospital, Medical University of Vienna, Waehringer Guertel 18-20, 1090 Vienna, Austria; 2 Department of Orthopedic Surgery and Traumatology, Evangelisches Krankenhaus, Hans-Sachs-Gasse 10-12, 1180 Vienna, Austria; 3 Karl Landsteiner Institut für Orthopädische Chirurgie, Karl Landsteiner Gesellschaft, Julius-Raab-Promenade 49/1, 3100 St. Pölten, Austria; 4 Adult Reconstruction & Joint Replacement Division, Hospital for Special Surgery, 535 East 70th Street, New York, NY 10021, USA; 5 Department of Orthopedic Surgery, University Hospital Ulm, Oberer Eselsberg 45, 89081 Ulm, Germany

**Keywords:** total knee arthroplasty, desired activity level, desired UCLA score, patients’ expectations

## Abstract

**Background/Objectives**: While most patients resume sports within one year after total knee arthroplasty (TKA), data on whether patients achieve their desired level of sports activity remain limited. This study aimed to evaluate the relationship between desired sports activity levels and postoperative outcomes after TKA and to identify factors associated with achieving the desired activity level. **Methods**: This retrospective cohort study included 280 patients (63.9% female; mean age, 65.7 years) who underwent primary TKA with a mean follow-up of 28.2 months. The University of California and Los Angeles (UCLA) activity score was used pre- and postoperatively to assess the desired and the achieved activity level. **Results:** The mean UCLA activity score improved significantly after surgery (from 4.6 to 5.6; *p* < 0.001). However, the mean preoperative desired UCLA score was significantly higher than the mean postoperative achieved UCLA score (7.1 vs. 5.6; *p* < 0.001). Overall, 34.6% of patients reached their desired activity level. These patients demonstrated a significantly lower preoperative desired UCLA score (6.1 vs. 7.6; *p* < 0.001) and a higher postoperative achieved UCLA score (6.7 vs. 5.0; *p* < 0.001) compared with patients who did not. Male sex, higher preoperative UCLA scores, and lower preoperative desired UCLA scores were identified as independent predictors of achieving the desired activity level. **Conclusions**: The present study demonstrated that despite excellent Knee Society function and WOMAC scores only 1/3 of patients reach their sport-related desired activity level. Surgeons must ensure that they communicate realistic expectations to patients prior to surgery, in order to avoid dissatisfaction that may arise from unmet expectations.

## 1. Introduction

The annual number of primary total knee arthroplasties (TKAs) in the United States is projected to reach 1.26 million by 2030, representing an 85% increase compared with 2014. While this rise is commonly attributed to an aging population, a substantial increase has also been observed among patients younger than 65 years [1]. This younger patient cohort is generally more physically active and reports higher expectations regarding sports participation and return to sports (RTS) following TKA [2,3]. Previous studies have reported that up to 84% of patients return to sports within one year after TKA, with approximately 70% achieving this outcome within the first six months [4].

Postoperatively, patients typically demonstrate higher levels of physical activity and are able to participate in a broader range of sports compared with their preoperative state. This improvement is mainly observed in low- and moderate-impact activities, which constitute the majority of sports resumed after TKA, whereas only a small proportion of patients return to high-impact sports [5]. Due to limited long-term data on implant survival in highly active patients, recommendations regarding participation in high-impact sports remain cautious, despite encouraging mid-term outcomes reported in the literature [6,7,8,9,10].

Although numerous studies have investigated RTS after TKA, there is currently no standardized definition of RTS, and most studies do not consider whether patients return to their preoperatively desired level of sports activity. As a result, patients may be classified as having successfully returned to sports despite failing to achieve their personal activity goals, which may negatively affect postoperative satisfaction. Therefore, evaluating whether patients achieve their desired level of sports activity is of particular clinical relevance. Previous research has also demonstrated that achieving preoperative goals is strongly associated with higher patient satisfaction following TKA [11]. Consequently, both patient expectations and the ability to meet these expectations play a crucial role in postoperative outcomes. Several factors may influence the desired activity level and the likelihood of achieving it, including age, sex, and other patient-specific characteristics [10]. In addition, pain levels and postoperative range of motion (ROM) have been identified as important contributors to functional outcomes following TKA [12,13].

Therefore, this study aimed to evaluate the relationship between the preoperative desired and actual postoperative sports activity levels following TKA, and to identify factors associated with achieving the desired activity level. We hypothesized that (1) the majority of patients would not achieve their preoperatively desired activity level after TKA, and (2) patient-related factors such as age, sex, and preoperative activity level would significantly influence both the desired activity level and the likelihood of achieving it postoperatively.

## 2. Materials and Methods

This retrospective cohort study evaluated patients following primary total knee arthroplasty (TKA) who were stratified according to whether they achieved their preoperatively desired University of California and Los Angeles (UCLA) activity score. A consecutive series of patients with documented pre- and postoperative desired and current activity levels, with a minimum follow-up of 12 months, was reviewed.

The original cohort consisted of 341 TKAs. However, in patients who underwent bilateral TKA during the study period, only the more severely affected knee was included in the analysis, as it represented the primary limitation to physical activity. This resulted in 61 knees being excluded from the study. The final cohort consisted of 280 TKAs (179 female, 63.9%) with a mean age of 65.7 years and a mean body mass index (BMI) of 30.1 kg/m^2^. The mean follow-up duration was 28.2 months, and all patients were operated on between 2010 and 2014. Ethical approval was obtained from the local institutional review board prior to study initiation (#2016-0354).

Preoperatively, patients were asked to report their current level of sports activity as well as the level of activity they desired to achieve following surgery. At follow-up, patients were again asked to report their current activity level to determine whether their preoperative expectations had been met. In addition, patients were asked to reassess their desired activity level at follow-up to evaluate potential changes in expectations over time. Current and desired levels of sports activity were quantified using the UCLA activity score, which ranges from 1 to 10 [14,15]. Information on desired and actual levels of sports activity, along with the other parameters used in this study, was routinely collected as part of standard clinical care during outpatient visits. This information was then extracted from the medical records retrospectively for analysis.

Extracted clinical data also included the Knee Society Score (KSS) comprising the knee score and functional score, both ranging from 0 to 100 [16], the Western Ontario and McMaster Universities Osteoarthritis Index (WOMAC) with a Likert scale ranging from 0 to 96 [17] and the visual analogue scale (VAS) [18] for the evaluation of pre- and postoperative pain levels. The preoperative assessments were conducted the week before surgery.

Pre- and postoperative mechanical alignment was assessed by measuring the hip–knee–ankle (HKA) angle on standardized full weight-bearing standing hip-to-ankle radiographs, as previously described by Cooke et al. [19]. The assessment was conducted by two blinded orthopedic residents with three and five years of clinical experience. All radiographic measurements were performed using a picture archiving and communication system (PACS) and a commercial planning software (Sectra IDS7; Version 26.1.21.8030; Sectra, Linköping, Sweden). Intra- and interobserver reliability were calculated to ensure the comparability of measurements, achieving excellent results (0.90 and 0.96).

All surgical procedures were performed by a single high-volume arthroplasty surgeon (FB) at a specialized arthroplasty center using hypotensive epidural anesthesia and a tourniquet. A standard medial parapatellar approach with a femoral first technique was employed in all cases, with the surgical goal of achieving a neural mechanical axis.

All patients received a posterior-stabilized (PS) total knee system with high-flexion design inlays. In cases of residual medial or lateral laxity following comprehensive soft tissue release, a constrained insert was used. Four different implant systems were utilized during the study period: (1) Genesis II SPC Total Knee System/Legion (Smith&Nephew, Memphis, TN, USA), (2) Balanced knee system (Ortho Development, Draper, UT, USA), (3) NexGen Legacy LPS-Flex Knee (Zimmer, Warsaw, IN, USA) and the (4) Sigma Total Knee System (DePuy Synthes, Warsaw, IN, USA). Changes in implant systems reflected surgeon preference over time and were not related to patient-specific clinical characteristics. Patellar resurfacing was performed in all cases, and all components were cemented.

All patients followed a standardized rehabilitation protocol. There were no documented drop-outs, and completion of the rehabilitation program was required prior to clearance for return to sports. The protocol focused on progressive range of motion, icing, gait training and biking. While progression through the protocol was individualized based on functional milestones (e.g., achieving full extension), the overall duration of eight to twelve weeks and structure of rehabilitation were comparable across patients.

## 3. Statistical Analysis

Normality of continuous variables was assessed using the Shapiro–Wilk test. As preoperative and postoperative actual and desired UCLA activity scores were not normally distributed, comparisons were performed using the Wilcoxon signed-rank test. Continuous variables are reported as mean ± standard deviation or as median with range, as appropriate. Between-group comparisons were conducted using the unpaired t-test for normally distributed variables and the Mann–Whitney U test for non-normally distributed variables. Categorical variables were compared using the chi-square test or Fisher’s exact test, as appropriate.

A multiple linear regression analysis using the standard Enter-Method was performed to evaluate the association between the preoperative desired UCLA score and patient-related factors, including age, sex, BMI, ROM, and clinical outcome scores. In addition, a binary logistic regression analysis was employed to assess the influence of the same variables on the likelihood of achieving the preoperatively desired UCLA score postoperatively.

A *p*-value < 0.05 was considered statistically significant, and a *p*-value < 0.001 was considered highly significant. All statistical analyses were performed using IBM SPSS Statistics for Mac OS X, Version 29.0 (IBM Corp., Armonk, NY, USA). Intra- and interobserver reliability of the radiographic measurements of the HKA was assessed using the intraclass correlation coefficient (ICC). ICC values were interpreted according to commonly accepted thresholds: values < 0.50 were considered poor, 0.50–0.75 moderate, 0.75–0.90 good, and >0.90 excellent reliability [20]. The HKA was measured on 20 randomly selected preoperative and postoperative radiographs by two independent, blinded orthopedic residents with three and five years of clinical experience.

## 4. Results

**Figure 1:**
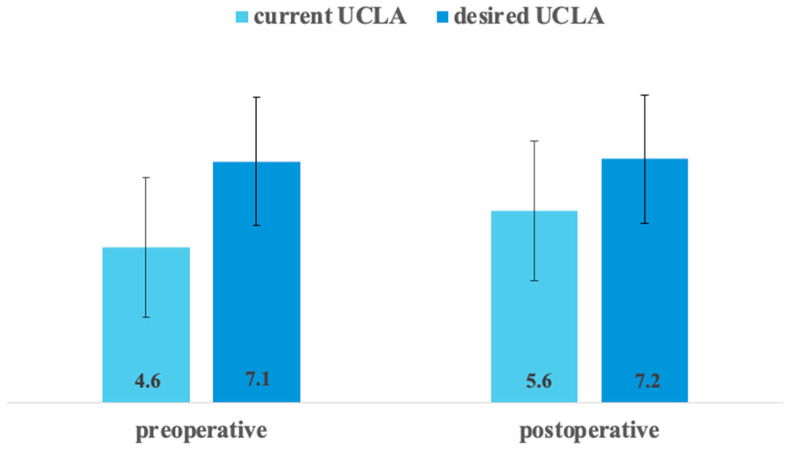
Comparison between current and desired UCLA score.

The mean UCLA activity score improved significantly following surgery, increasing from 4.6 preoperatively to 5.6 postoperatively (*p* < 0.001). Preoperatively, UCLA activity levels 1–3 accounted for 31.8% of patients, which decreased to 9.4% at follow-up (Table 1). However, the mean preoperative desired UCLA score was significantly higher than the mean postoperative actual UCLA score (*p* < 0.001; Figure 1).

A subgroup of patients who reached their preoperative desired UCLA score was identified by individually comparing each patient’s preoperative desired UCLA score with their postoperative actual UCLA score. Patients whose postoperative current UCLA score matched (68 patients) or exceeded (29 patients) their individual preoperative desired score were included in this subgroup. Notably, 34.6% of patients achieved their preoperatively desired activity level. A comparison of the two subgroups revealed no statistically significant disparities with respect to demographic and clinical variables. Detailed data of the subgroups are presented in Table 2.

Subsequent analyses revealed that both subgroups demonstrated comparable preoperative actual UCLA scores. However, patients who did not achieve their desired activity level reported significantly higher preoperative expectations (mean desired UCLA score: 7.6 vs. 6.1) and attained significantly lower postoperative activity levels (mean postoperative UCLA score: 5.0 vs. 6.7). In addition, patients who achieved their desired activity level tended to have lower preoperative and better postoperative patient-reported outcome measures. Detailed PROM data are shown in Table 3.

**Figure 2:**
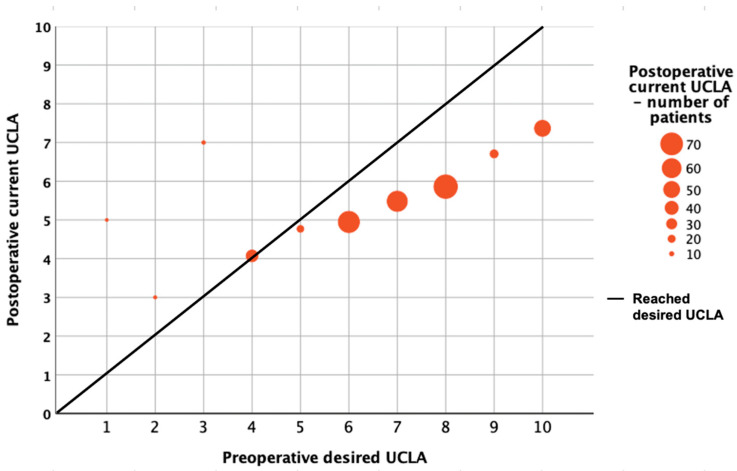
Mean postoperative current UCLA score of all patients with the same preoperative desired UCLA score.

Patients with higher preoperative desired UCLA scores achieved higher mean postoperative activity levels but were more likely to fall short of their individual expectations. Patients with a preoperative desired UCLA score of 10 demonstrated the largest discrepancy between expected and achieved activity levels, with a mean postoperative UCLA score of 7.3 (Figure 2). In contrast, patients with a preoperative desired UCLA score of 6 achieved a mean postoperative UCLA score of 4.9.

Postoperative expectations were also evaluated. A patient was considered to have achieved their postoperative activity goal if the postoperative desired UCLA score matched the postoperative actual UCLA score. Based on this definition, 94 of 280 patients (33.6%) achieved their postoperative desired activity level.

Regression analyses demonstrated that younger age, male sex, and a higher preoperative actual UCLA score were significantly associated with higher preoperative desired UCLA scores. Furthermore, male sex, a higher preoperative actual UCLA score, and a lower preoperative desired UCLA score were identified as significant predictors of achieving the preoperatively desired activity level. Detailed regression results are provided in Table 4.

## 5. Discussion

The principal finding of the present study was that, despite a significant overall improvement in postoperative activity levels and excellent clinical outcomes as reflected by high KSS scores, patients’ postoperative actual UCLA scores remained significantly lower than their preoperatively desired UCLA scores. This indicates that, although functional improvement is commonly achieved after TKA, many patients do not reach the level of sports activity they expect prior to surgery. Subgroup analysis further demonstrated that patients who achieved their desired activity level reported significantly lower preoperative desired UCLA scores (6.1 vs. 7.6, *p* < 0.001) and higher postoperative actual UCLA scores (6.7 vs. 5.0, *p* < 0.001), suggesting that more modest expectations facilitate goal attainment.

Interestingly, the subgroups did not differ regarding the intensity of pre- and postoperative pain. This finding suggests that pain alone may not be the primary limiting factor for postoperative sports participation. Patients may tolerate residual pain in order to remain active, whereas other factors—such as expectations, motivation, or functional capacity—may exert a stronger influence. The importance of realistic expectation setting is further highlighted by the fact that patients with higher preoperative expectations achieved higher absolute postoperative activity levels but were more likely to fall short of their desired goals.

The mismatch between preoperative expectations and postoperative activity levels may be explained by a combination of physical, psychological, and behavioral factors. Residual pain, limited range of motion, or persistent strength deficits may only become relevant in higher-level activities. The desire to do higher physical activities might be not founded based on the patients’ abilities but could be a more general desire, and other factors, like weight, general health or ability to do certain sports might be reason that the theoretic desire for a sport is not achieved. Additionally, fear of injury and decreased longevity, and surgeon-imposed activity limitations may further limit sports participation. These factors may contribute to unmet expectations even when objective functional outcomes in this group appear favorable.

Harbourne et al. [21] conducted a study that evaluated 420 unicondylar knee arthroplasties (UKAs) and 575 TKAs to identify predictors of a patient’s inability to return to their desired activity within twelve months post-surgery. Patients were permitted to select up to three activities which they wished to resume postoperatively. They were subsequently questioned about their ability to return to these activities. Out of the 575 TKAs, 339 patients (59%) returned to their desired activities, which were primarily low-impact activities such as walking, cycling or gardening. They found that predictive factors for not returning to the desired activities after TKA were a lower preoperative Oxford Knee Score (OKS) and lower preoperative EuroQol-5D-3L (EQ-5D) score, which is a self-reported measure of a patient’s general health status. These results do not align with our observations, as our study revealed that the majority of patients experienced an inability to return to their preoperatively desired level of activity. Moreover, their findings did not support the hypothesis that the preoperative UCLA score could serve as a predictor for return to the desired level of sport, whereas our study demonstrated that patients with higher preoperative UCLA scores were more likely to resume their desired activities. This is consistent with the current literature, as Huch et al. [22] described RTS rates of 36% and 81%, regarding whether the preoperative sports participation percentage was chosen as participated “in some point during life” or as participated “at time of surgery”. This demonstrates that patients who were still active in a sport at the time of surgery are more likely to return to it [23].

A meta-analysis conducted by Konings et al. [5] reviewed five studies that used the UCLA score to compare the pre- and postoperative level of sport in TKA patients. Across these five studies, 2436 patients showed a mean improvement of 0.7 ± 0.2 in their postoperative UCLA score, which is consistent with our findings. However, patients may not perceive this improvement as positive, but rather as frustrating, because of the lack of progress compared to their expectations [24]. Therefore, although we believe that it is crucial to evaluate the improvement of the actual sport level in patients, we would like to emphasize the importance of relating the improvement in the level of sport to the patients’ desires. Lützner et al. [11] identified the most important expectations of TKA patients. 306 out of 352 patients (87%) classified an improvement in sport as a mandatory goal. However, of the 16 key expectations, this was the least fulfilled expectation, with 11% of patients reporting it as “not fulfilled” and 42% of patients reporting it as “partially fulfilled”. When comparing highly satisfied and not fully satisfied TKA patients, highly satisfied patients had a significantly higher proportion of key expectations “met” or “exceeded”. Therefore, meeting a patient’s expectations regarding their ability to participate in sports is one of the key factors in overall patient satisfaction [25]. We know that most patients return to low- and moderate-impact activities after TKA [5]. This is why it is crucial to assess and adjust patients’ expectations before surgery, in order to avoid dissatisfaction due to unmet expectations [26].

Regarding determinants of preoperative expectations, younger age, male sex, and higher preoperative activity levels were associated with higher desired UCLA scores. These findings are consistent with prior studies reporting that younger, more active male patients exhibit the highest expectations concerning sports participation following TKA [2,3,27]. The strong influence of male sex on both preoperative expectations and the ability to reach them may reflect differences in baseline activity levels, motivation, or societal expectations regarding sports participation. However, as demonstrated in the findings of this study, patients who initially set the highest expectations for their surgical outcomes exhibit a greater discrepancy between their postoperative results and their preoperative expectations, despite the tendency to achieve higher postoperative UCLA scores. Therefore, it is imperative to adjust the expectations of this demographic group prior to surgery to mitigate the risk of postoperative dissatisfaction stemming from unmet expectations. As the regression analyses were primarily intended to identify associations rather than to establish a predictive model, measures of explained variance were not emphasized. It is likely that a considerable proportion of variability is added by psychosocial and contextual factors that were not available in this retrospective dataset.

From a clinical perspective, structured expectation counseling tools, such as expectation questionnaires or tailored goal-setting strategies, may help bridge the gap between desired and achievable activity levels after total knee arthroplasty. Surgeons could incorporate standardized expectation assessment tools during preoperative consultations to identify patients with high or potentially unrealistic activity goals. Counseling should address individual physical abilities, common limiting factors such as obesity, poor overall health, persistent muscle strength deficits, residual pain, and restricted range of motion, as well as psychological barriers including fear of injury and reduced confidence in the operated knee. Clear, individualized guidance regarding postoperative activity restrictions and realistic timelines for return to sport may further support goal alignment. In addition, integrating patient motivation and activity preferences into shared decision-making may facilitate tailored rehabilitation strategies and improve postoperative satisfaction.

Additionally, advances in implant technology and sports-specific rehabilitation programs may further improve postoperative outcomes after TKA. Modern implant designs and materials may better accommodate higher activity demands, while individualized and sports-oriented rehabilitation approaches could support a more effective return to activity. Future research should assess whether these developments help patients achieve their desired activity levels.

Our study has several limitations due to its retrospective nature. (1) We only included patients for whom pre- and postoperative current and desired UCLA scores were available, which might result in a selection bias. (2) PROMs are a subjective method of assessment and may be influenced by individual perception and reporting bias. (3) Another limitation is the heterogeneity of the follow-up period. However, as Meena et al. [28] showed that there is no significant improvement in activity level after one year after surgery, the different lengths of follow-up periods do not significantly affect our results, because we had a minimum follow-up period of one year. (4) The study is also limited by its relatively old data, but, as several studies have shown that there has been little progress in patient satisfaction in recent years [29,30], our statement that the patient’s desired level of sport needs to be assessed preoperatively, to avoid unrealistic expectations affecting patient satisfaction, is still valid. (5) We decided to include patients who had both knees replaced during our evaluation period. However, since bilateral surgery had no significant effect on the patient’s ability to achieve their preoperative desired UCLA score, and we only included the worse knee to avoid having two activity scores for one patient, the inclusion of bilateral patients does not affect our results. (6) Although four different implant systems were used, implant selection was not based on patient characteristics. Nevertheless, potential effects of implant design on sports participation cannot be excluded. (7) When patients are asked for a desired activity level, that could trigger them to document a desire rather than a realistic assessment of abilities and actual physical possible levels of activities; the fact that those goals are not met could also be related to other factors not related to the knee replacement.

## 6. Conclusions

Overall, despite excellent Knee Society function and WOMAC scores, only a third of patients achieved their preoperatively desired level of sports activity at follow-up. Thus, despite a significant improvement in mean postoperative function and activity levels, sport-related satisfaction may be limited due to unmet expectations. These findings underscore the importance of assessing not only actual activity levels but also preoperative desired activity levels when evaluating outcomes after total knee arthroplasty. Incorporating structured expectation-setting protocols, patient education, and individualized goal-setting strategies into preoperative counseling may help align expectations with realistic postoperative outcomes and improve patient satisfaction. Future studies should investigate structured expectation-management programs and sports-oriented rehabilitation protocols to optimize postoperative activity levels and satisfaction.

## Figures and Tables

**Table 1:** Comparison of all patients at each UCLA level pre- and post-surgery.

UCLA Level	Sport Level	Percentage of Patients Prior to Surgery	Percentage of Patients After Surgery
**10**	Regularly participates in high impact sports such as jogging, tennis, skiing, acrobatics, ballet, heavy labor, or backpacking	**2.5%**	**4.2%**
**9**	Sometimes participate in high impact sports	**1.1%**	**5.3%**
**8**	Regularly participate in very active events such as bowling or golf	**6.4%**	**8.6%**
**7**	Regularly participate in active events such as bicycling	**7.9%**	**10.8%**
**6**	Regularly participate in moderate activities such as swimming and unlimited housework or shopping	**11.4%**	**22.9%**
**5**	Sometimes participate in moderate activities	**17.1%**	**14.6%**
**4**	Regularly participate in mild activities such as walking, limited housework, and limited shopping	**21.8%**	**24.3%**
**3**	Sometimes participate in mild activities	**12.5%**	**2.9%**
**2**	Mostly inactive; restricted to minimal activities of daily living	**17.9%**	**6.1%**
**1**	Wholly inactive; dependent on others; cannot leave residence	**1.4%**	**0.4%**

**Table 2:** Comparison of demographic and clinical data between patients who achieved or exceeded their preoperative desired UCLA score and patients who did not meet their expectations.

Parameter	Desired UCLA Achieved	Range	SD	Desired UCLA Not Achieved	Range	SD	*p* Value
**Patients (*n*)**	97	N/A	N/A	183	N/A	N/A	
**Sex, female:male (*n*)**	59:38	N/A	N/A	120:63	N/A	N/A	0.436 ^
**Mean age (years)**	66.4	27.3 to 86.2	10.21	65	37.9 to 87.0	9.43	0.233 *
**Mean BMI (kg/m^2^)**	29.4	19.4 to 45.7	5.32	30.8	17.1 to 60.5	6.22	0.121 †
**Mean follow-up (months)**	27.9	12.0 to 62.0	12.38	28.5	12 to 56	12.02	0.663 †
**Mean preoperative HKA (degree)**	−1.7	−22.2 to 23.6	10.07	−3.8	−40 to 25.4	9.91	0.115 †
**varus:neutral:valgus (*n*)**	59:5:33	N/A	N/A	119:9:55	N/A	N/A	
**Mean postoperative HKA (degree)**	−0.3	−8.3 to 7.9	2.72	−0.9	−10.3 to 5.8	2.8	0.283 †
**varus:neutral:valgus (*n*)**	34:37:26	N/A	N/A	69:77:37	N/A	N/A	
**Preoperative ROM (degree)**	104.4	0 to 130	24.43	109.3	50 to 140	17.19	0.185 †
**Postoperative ROM (degree)**	123.1	70 to 130	7.55	123	90 to 140	6.97	0.717 †

^ Fishers exact test; * Unpaired *t*-test; † Mann–Whitney U test; SD, standard deviation; N/A, not applicable; BMI, body mass index; ROM, range of motion; HKA, hip–knee–ankle angle.

**Table 3:** Comparison of patient-reported outcome measures of patients who reached their preoperative desired UCLA score and those who did not.

Parameter	Desired UCLA Achieved	SD	Desired UCLA Not Achieved	SD	*p* Value
**preoperative actual UCLA**	4.5	2.08	4.6	2.03	0.842 †
**preoperative desired UCLA**	6.1	2.01	7.6	1.58	**<0.001 †**
**postoperative actual UCLA**	6.7	1.93	5	1.75	**<0.001 †**
**postoperative desired UCLA**	7.3	1.68	7.1	1.76	0.358 †
**preoperative VAS**	6.9	2.34	7	2	0.716 †
**postoperative VAS**	1.6	1.91	2.1	2.31	0.154 †
**Preoperative KSS objective**	40	20.8	45.5	19.46	**0.046 †**
**Postoperative KSS objective**	93.8	6.84	92	8.51	0.123 †
**Preoperative KSS function**	52.6	18.06	57	17.62	0.166 †
**Postoperative KSS function**	94.5	11	93.4	12.89	0.607 †
**Preoperative WOMAC**	43	17.62	43.3	15.63	0.903 *
**Postoperative WOMAC**	12.6	13.75	18.1	15.62	**0.001 †**

* Unpaired *t*-test; † Mann–Whitney U test.

**Table 4:** Analysis of potential factors influencing the preoperative desired UCLA score and the ability to reach the preoperative desired UCLA score.

Factors	Preoperative Desired UCLA (a)		Desired UCLA Achieved (b)	
	Estimate	*p* Value	Odds-Ratio	*p* Value
**Age**	−0.042	**<0.001**	−0.015	0.426
**BMI**	0.022	0.207	−0.025	0.422
**Gender (male)**	0.448	**0.038**	1.025	**0.006**
**preoperative ROM**	0.009	0.128	−0.002	0.889
**preoperative VAS**	0.033	0.530	−0.072	0.412
**preoperative KSS**	0.009	0.158	0.002	0.843
**preoperative KSS Function**	0.005	0.433	−0.020	0.109
**preoperative WOMAC**	0.010	0.172	0.006	0.66
**preoperative current UCLA**	0.471	**<0.001**	0.393	**<0.001**
**preoperative desired UCLA**	N/A		−0.803	**<0.001**
**postoperative ROM**			0.022	0.392
**follow-up**			−0.003	0.847
**bilateral surgery**			−0.356	0.401

(a) multiple linear regression; (b) logistic regression; N/A, not applicable.

## Data Availability

The original contributions presented in this study are included in the article. Further inquiries can be directed to the corresponding author(s).
